# The Effect of Surgical Intervention of Endometriosis to CA-125 and Pain

**DOI:** 10.21315/mjms2020.27.6.2

**Published:** 2020-12-29

**Authors:** Abdul Kadir Abdul Karim, Nor Haslinda Abd Aziz, Reena Rahayu Md Zin, Norfilza Mohd Mokhtar, Mohamad Nasir Shafiee

**Affiliations:** 1 Department of Obstetrics & Gynaecology, Faculty of Medicine, Universiti Kebangsaan Malaysia, Cheras, Kuala Lumpur, Malaysia; 2 Department of Pathology, Faculty of Medicine, Universiti Kebangsaan Malaysia, Cheras, Kuala Lumpur, Malaysia; 3 Department of Physiology, Faculty of Medicine, Universiti Kebangsaan Malaysia, Cheras, Kuala Lumpur, Malaysia

**Keywords:** endometriosis, CA-125, visual analogue scale, surgery

## Abstract

Endometriosis is an inflammatory condition characterised by the presence of endometrial growth beyond the uterine cavity. It is a debilitating disease requiring multiple modalities of treatment. In considering surgery as the option of treatment, the benefits should outweigh the risk. Besides direct surgical risk, intervention may lead to a reduction of ovarian reserve, in addition to premature menopause and low fecundity. To date, there is an inconclusive evidence to support any specific parameters in monitoring disease progression following surgical intervention. Serum cancer antigen (CA)-125 is expressed by coelomic epithelium and has been extensively studied as a biomarker for endometriosis. Elevated expression of CA-125 has been shown in endometrial tissues and the marker increased indirectly from peritoneal irritation that accompanies an extensive form of endometriosis. Additionally, the visual analogue scale (VAS) scores have been used as an objective measurement for measuring pain, especially in a complex disease such as endometriosis. This review aims to consolidate a series of clinical trials that utilised CA-125 level and VAS score as tools for monitoring patients undergoing surgery for endometriosis.

## Introduction

Endometriosis is a benign condition characterised by the presence of endometrial cells in the extrauterine cavity, which potentiates chronic inflammation. It commonly involves peritoneum and pelvic organs, with extra-abdominal locations are also being reported (1). The disease can be asymptomatic and could be discovered accidentally during laparoscopic surgery. The most typical symptoms include chronic pelvic pain that could last for 6 months or longer, dysmenorrhea and dyspareunia, which is pain occurring during coitus. Numerous theories have been proposed for the pathogenesis of endometriosis, such as retrograde menstruation, immunological response and progesterone resistance, with the presence of underlying perturbation of progesterone receptor isoforms ratio (2). Medical treatment has been shown to have varying success (3), while surgical treatment carries operative risk and may impair future fecundity.

The gold standard for diagnosis of endometriosis is by visual inspection, preferably by histological confirmation, thus requiring surgery with its added risks. The use of molecular biomarkers as a diagnostic tool in endometriosis remains inconclusive, albeit those biomarkers’ role in prognostication of the disease has so far never been clinically validated and broadly utilised. Hence, this review aims to consolidate evidence of the usefulness of cancer antigen (CA)-125 levels and pain score using visual analogue scale (VAS) at pre- and post-surgery for endometriosis, as the major predictive outcome.

## Methods

This review was performed as part of our molecular research on the correlation of serum CA-125 and pain scores on progesterone receptor isoform perturbations in patients with endometriosis, which was approved by our institutional ethical committee (FFF-2017-045). The available evidence for serum CA-125 and VAS scores concerning endometriosis surgery was reviewed. A literature search was performed using two databases, using MEDLINE on OVID and Scopus. The terms ‘Endometriosis’ and ‘CA125’ and ‘surg*/operati*’ were used for presence in the title, abstracts and keywords. Limits were set to the last three decades from 1990 to March 2020. A similar search was repeated with the term ‘visual analogue scale/VAS’ replacing ‘CA125’. Original studies of clinical trials of surgery on endometriosis patients involving the measurements of serum CA-125 and VAS were selected and reviewed. Relevant clinical papers that were cited in these articles were also scrutinised to ensure earlier critical information was not omitted. Related literature concerning the subjects was also analysed for completeness.

## Results

A total of 429 potentially relevant articles were retrieved from the search regarding endometriosis surgery in relation to CA-125 with 176 from the Scopus and 253 from MEDLINE. A total of 13 duplicates were then removed. Review articles, case reports and literature from books were excluded. A total of six articles were deemed relevant to our objective. Full-text articles were obtained for five, with only an English abstract was available for an original article published in China. An article was added from our preliminary search of the topic, which made it a total of seven articles were reviewed. A similar search for literature regarding endometriosis surgery in relation to VAS returned with a total of 324 articles with 43 from Scopus and 281 from MEDLINE. A total of 12 duplicates were removed. Review articles, case reports and literature from books were then excluded. A total of nine articles met the inclusion criteria and were deemed relevant to our objective. The full manuscripts were obtained for further review.

## Discussion

### The Effects of Surgery for Endometriosis on Serum CA-125 Level

CA-125, which is the glycoprotein expressed by coelomic epithelium, is an extensively studied marker. The previous study had shown that elevated levels of pre-operative serum CA-125 were recorded in patients with moderate to severe endometriosis compared to control (4). Furthermore, immunocytochemical studies indicated that CA-125 is present in the endometriotic lesion. The elevation observed in serum CA-125 and its presence in endometriotic lesions led to investigations as a diagnostic tool. However, the pathogenesis of endometriosis itself is still unclear; hence it is not surprising that a recent meta-analysis has shown that CA-125 is not useful for diagnostic purposes (5).

There are two postulations on how endometriosis causes a rise in the serum CA-125 level. The first is a higher concentration of antigen at the cell-surface levels in endometriotic lesions compared with normal endometrium. Secondly, it is caused by the inflammation that commonly accompanies endometriotic lesion (4). A subsequent study showed that endometriosis is not a source of elevated CA-125 level, but rather the peritoneal irritation that frequently accompanies extensive forms of the disease could have a significant role in causing the perturbation (6). On this basis, serum CA-125 may be used for monitoring the degree of peritoneal irritation due to endometriosis. Nonetheless, the CA-125 role in monitoring the disease has not been established.

Seven articles were included in the final review, as summarised in Table 1.

None of the articles found by a search in the two databases had a primary objective of comparison between the pre- and post-operative serum CA-125 levels in an endometriosis cohort. During an initial exploration of a search engine, a study was found to specifically look at the effects of surgery on endometriosis patients on serum CA-125. The study by Kokeb et al. (7) was a cross-sectional study conducted in an Asian population of a third world country. A total of 100 patients with endometriosis were included; however, the stages of the disease were not mentioned. The authors found that there was a highly significant drop of mean serum CA-125 level from pre- to post-operation (60.5 IU/mL to 27.5 IU/mL). Unfortunately, there was neither a control group nor mention of the actual time point for post-surgery when the levels were taken. Despite the shortcomings of the study, it was the only study that specifically investigated the effects of surgery for endometriosis on serum CA-125 level.

All seven studies had pre-surgery serum CA-125 levels. The levels varied between studies with two studies showing normal levels (8, 9) and five publications showing elevated levels (7, 10–13). Yet, comparison to a control group was only available in three studies. Nevertheless, only one study (8) performed an analysis comparing the control and endometriosis groups during pre-surgery, which did not show any statistical significance. This, however, was in an asymptomatic cohort of endometriosis patients. The other two studies, which had a control group, did not elaborate on statistical analysis for differences (9, 11). Thus, the evidence suggested that pre-surgery levels in patients with endometriosis varied. In the minimal to a mild stage of the disease, the levels for CA-125 were similar to controls.

Only three studies had post-surgery levels of serum CA-125 (7, 10, 11). However, the time of measurement of post-operative values varied from 24 h to 6 months. The differences in the time points could explain the elevated levels of serum CA-125 as observed in Ruan et al. (10), which measured at earlier time point but normal levels are reported by Kokeb et al. (7), which measured serum CA-125 level after several weeks of operation.

Only the three studies mentioned above had measured pre- and post-surgery levels in their endometriosis cohort (7, 10, 11). All three showed reductions in the serum CA-125 levels after surgery. Ruan et al. (10) reported a decrease from 55.8 U/mL to 34.3 U/mL at 24 h; however, no statistical analysis for the difference was published. The study by Kokeb et al. (7) had shown a significant drop from 60.5 IU/mL to 27.5 IU/mL; however, the time point for post-surgery was not revealed. The publication by Liu et al. (11) had shown a significant drop after 4 weeks, but no values were available due to information available was only from an English abstract. Therefore, all evidence points towards the improvement of serum CA-125 up to 12 weeks after the surgery for endometriosis. Although it has been shown by Cochrane meta-analysis (5) that serum CA-125 is not useful as a diagnostic tool for endometriosis, there is a potential use if monitoring is required after surgical intervention.

Overall the articles reviewed here varied in quality. The main problem was the lack of studies comparing pre- and post-surgery as the primary outcome. All studies had pre-surgery serum CA-125 levels, but only three out of seven had post-surgery levels for comparison. Of the seven studies, only three had a control group, yet there were variations in the methods measuring CA-125 amongst the studies.

### The Effects of Surgery for Endometriosis on Pain

One of the main symptoms of endometriosis is pain. A systematic review (14) found that VAS is the most frequently used scale, which can provide both clinicians and researchers with tools to evaluate treatment response for endometriosis-related pain. Improvement in the pain score may reflect the surgical disruption of the neurogenic inflammation, hyperalgesia and dysreflexia of various pelvic viscera (15). Nine studies were included in our final analysis.

It is difficult to compare the VAS scores as the studies reviewed in the literature reported using both mean and median scores. Furthermore, the type of VAS used varied. Seven of the studies described using a 10-point VAS for measurement (16–22). Another two used a 10 cm linear scale (23) and an 11-point scale (24), respectively. To compound matters, the classification of pain also varies from just pelvic pain in two studies (17, 23) to a breakdown of the type of pain, which were dysmenorrhea, dyspareunia and non-menstrual pelvic pain or chronic pelvic pain in five studies (16, 18–20, 24). The use of just pelvic pain, which was inclusive of all parameters, would have been more advantageous as the patients may not have resume or cease having coitus or menstruation during follow-up.

The time points when the VAS scores were documented post-surgery also varied between studies from 1 month up to 60 months. There were three studies (18, 19, 23), which recorded VAS scores at 3 months. Out of those, only Sutton et al. (23) measured pelvic pain VAS score using a median score, which showed a non-statistically significant improvement. A possible reason that the result did not reach significance was the fact that 90% of their cohort was in stage I–II. The other two studies also showed improvement in dysmenorrhea, dyspareunia and pelvic pain; however, no statistical analysis for the difference was published. On the other hand, four studies showed a statistically significant drop in pain score (16, 20, 21, 24). These studies had taken their VAS scores between 6 to 60 months. Most of these studies agreed that there was significant pain improvement achieved at 6 months post-surgery.

All of the nine studies included in this review used the revised American Society of Reproductive Medicine (rASRM) staging for endometriosis (25). In terms of the cohort of endometriosis patients, those with a majority of patients in stage I–II (16, 19, 20, 23) and those with a majority in stage III–IV (17, 24), all showed a reduction in VAS scores. However, only four studies (16, 19, 20, 23) showed a significant reduction. Although the evidence showed a marginal and inconsistent association between endometriosis stage and the severity of pelvic symptoms (26), the studies reviewed suggest that pain improvement was noted regardless of the stage of the disease.

The studies in our review did not have any direct comparison between laparoscopic and open surgery in improving VAS scores. The majority of the studies in this review used a laparoscopic approach for surgery. The laparoscopic approach is, however, advisable as the previous meta-analysis has shown it to be effective. Evidence from Cochrane meta-analysis (27) suggests that laparoscopic surgery compared to diagnostic laparoscopy alone reduces pain associated with endometriosis in all stages of the disease. The summary of the clinical studies reviewed for the effects of endometriosis surgery on VAS scores is shown in Table 2.

There is a challenge in interpreting these studies due to the non-homogeneity of the methods. These include the design, type of surgery, post-surgery time points of VAS scores, method of reporting results and the description of pain. A positive note was that six out of the nine studies were reasonably recent, less than 10 years (16–20, 22). However, all studies were in agreement that there is an improvement in either the mean or median of the VAS scores after the surgeries, with three studies reported significant reduction (16, 20, 24).

## Conclusion

The myriad of pathways in the pathogenesis of endometriosis makes the diagnosis and monitoring of disease difficult. Surgery is one of the options of treatment for endometriosis, and a method to monitor the benefit of this modality is much sought after. Serum CA-125 has been shown to be non-beneficial as a tool for diagnosis. However, several studies have suggested the role of monitoring as similar patterns of improvement were observed in the serum CA-125 levels after surgery. Further studies with the primary outcome of improvement in serum CA-125 levels after surgery and reduced variables are necessary for confirmation. In addition to CA-125, studies have shown improvement in VAS scores after surgery and the improvement became significant after 3 months as pain is one of the main symptoms associated with endometriosis. Despite risk due to the surgery itself, endometriosis surgery should be recommended for the improvement of pain and serum CA-125 should be considered as a monitoring tool post-surgery.

## Figures and Tables

**Table 1:** Clinical trials utilising serum CA-125 in endometriosis patients undergoing surgery

Author	Year	Cohort (N)	Methodology	Measuring method	Outcome
Barbosa et al. (8)	2009	Asymptomatic patients for Tubal sterilization Endometriosis (13) Control (67)	Cross-sectional CA-125 pre-surgery	Not mentioned	Mean levels between groups not statistically significant (26.9 versus 28.3) U/mL
Salehpour et al. (9)	2009	Laparoscopic surgery Endometriosis (35) Control (25)	Cross-sectional CA-125 pre-surgery	Electrochemiluminescence immunoassay	Mean higher in endometriosis group (26.4 versus 12.6) IU/mL. (*P* < 0.05)
Ruan et al. (10)	2015	Endometriosis Open surgery (50) Laparoscopic (50)	Prospective randomised CA-125 pre-surgery and post-surgery (6 h, 12 h, 24 h)	Chemiluminescence	Mean for pre-surgery similar (55.7 versus 55.8) U/mL. 24 h post surgery (34.3 versus 37.5) U/mL
Karimi-Zarchi et al. (12)	2016	Symptomatic endometriosis (87)	Cross-sectional CA-125 pre-surgery	ELISA kit monoclonal (Roche, Germany)	Mean level elevated at 49.9 U/mL
*Liu et al. (11)	2016	Endometriosis (105) Control (100)	CA-125 pre-surgery and post-surgery (4 weeks, 6 months)	ELISA	Pre-operative mean higher in endometriosis group. Post-surgery at 4 weeks, it reduced significantly but still higher than control group.
Kokeb et al. (7)	2017	Endometriosis (100)	Cross-sectional CA-125 pre-surgery and post-surgery (time point not mentioned)	Not mentioned	Significant drop of mean (60.5–27.5) IU/mL
Li et al. (13)	2019	Laparoscopic surgery for endometrioma Recurrence (68) No recurrence (290)	Retrospective CA-125 pre-surgery	Not mentioned	Pre-operative mean Recurrence group 99.2 U/mL versus non-recurrence group 100.4 U/mL

Notes: ELISA = Enzyme-linked immunosorbent assay;

* Only English abstract available

**Table 2:** Clinical studies assessing the visual analogue scale score of endometriosis patients from pre- to post-surgery

Author	Year	Cohort (N)	rASRM Stage	Methodology/Time points	Outcome
Sutton et al. (17)	1994	Laparoscopic surgery Laser ablation and LUNA (32) Expectant (31)	90% cohort in stage I–II	Prospective randomised double-blinded. Time points post-surgery (3 and 6 months)	Median score both groups improved but not statistically significant
Abbott et al. (21)	2003	Laparoscopic excision surgery (135)	43% cohort in stage I–II	Prospective observational. Time points post-surgery (24 to 60 months)	Median score had significant drop for Dysmenorrhoea (9 to 3) Dyspareunia (7 to 3) Non menstrual pelvic pain (7 to 0)
Healy et al. (19)	2010	Laparoscopic surgery Ablative (49) Excision (54)	78% cohort in stage I–II.	Prospective randomised double-blinded. Time points post-surgery (3, 6, 9 and 12 months)	Mean score was reduced at 12 months for Dysmenorrhoea Dyspareunia Pelvic pain
Healy et al. (20)	2014	Laparoscopic surgery Ablative (49) Excision (54)	78% cohort in stage I–II	Prospective randomized double-blinded. Time points post-surgery (60 months)	Mean scores had significant reduction for Dysmenorrhoea Dyspareunia Pelvic pain
Ahmad et al. (22)	2017	Primary endometriosis surgery (280). Laparoscopic approach in 80%	94% cohort in stage I–II	Prospective cohort. Time points post-surgery (6 months)	Mean scores had significant reduction for Dysmenorrhoea (5.8 to 4.1) Dyspareunia (4.1 to 2.2) Non menstrual pelvic pain (4.9 to 3.2)
Alborzi et al. (16)	2017	Laparoscopic endometriosis surgery (1086)	17.5% cohort in stage I–II	Prospective observational. Time points post-surgery (6 months)	Mean scores had reduction (8.2 to 4.5) in 93% of patients.
Dobrokthova et al. (18)	2017	Laparoscopic endometriosis surgery Post-surgery (expectant) (20) Post-surgery (hormonal) (33)	Stage of disease not mentioned	Prospective randomised. Time points post-surgery (1, 3, 6 and 12 months)	Mean scores had reduction at all time points for both groups for Dysmenorrhoea Dyspareunia Chronic pelvic pain
Riley et al. (27)	2019	Laparoscopic endometriosis surgery Excision (37) Ablation (36)	All in Stage II–III	Prospective randomised. Time points post-surgery (6 and 12 months)	Ablation group Dyspareunia: significant improvement at 6 months Dysmenorrhoea: significant improvement at 6 and 12 months Excision group No significant difference at 6 and 12 months
Modarresi et al. (23)	2020	Laparoscopic cystectomy of endometrioma (79)	Stage of disease not mentioned	Cross-sectional. Time points post-surgery (6 months)	Mean scores had significant improvement Pelvic pain (5.0 to 1.1) Dsymennorhoea (8.0 to 3.2) Dyspareunia (1.8 to 0.4)

Notes: LUNA = laparoscopic uterine nerve ablation; rASRM = revised American Society for Reproductive Medicine

## References

[1] Pankratjevaite L, Samiatina-Morkuniene D (2017). A case report of thoracic endometriosis – a rare cause of haemothorax. Int J Surg Case Rep.

[2] Abdul Karim AK, Shafiee MN, Abd Aziz NH, Omar MH, Zin RRM, Mokhtar NM (2019). Perturbations in progesterone receptor isoforms in endometriosis. J Reprod Med.

[3] Abdul Karim AK, Shafiee MN, Abd Aziz NH, Omar MH, Abdul Ghani NA, Lim PS (2019). Reviewing the role of progesterone therapy in endometriosis. Gynecol Endocrinol.

[4] Barbieri RL, Niloff JM, Bast RC, Schaetzl E, Kistner RW, Knapp RC (1986). Elevated serum concentrations of CA-125 in patients with advanced endometriosis. Fertil Steril.

[5] Nisenblat V, Bossuyt PMM, Shaikh R, Farquhar C, Jordan V, Scheffers CS (2016). Blood biomarkers for the non-invasive diagnosis of endometriosis. Cochrane Database Syst Rev.

[6] Fedele L, Arcaini L, Baglioni A, Bianchi S, Menard S (1989). What is the source of elevated serum levels of CA 125 in patients with endometriosis?. Eur J Obstet Gynecol Reprod Biol.

[7] Kokeb S, Inayat K, Ahmad S, Tabassum A (2017). Decrease in level of CA-125 after surgical treatment of endometriosis. Gomal J Med Sci.

[8] Barbosa CP, Souza AMBd, Bianco B, Christofolini D, Bach FAM, Lima GRd (2009). Frequency of endometriotic lesions in peritoneum samples from asymptomatic fertile women and correlation with CA125 values. Sao Paulo Med J.

[9] Salehpour S, Sene AA, Mehrjerdi EK, Akhoond MR (2009). The correlation between serum and peritoneal fluid CA125 level in women with pelvic endometriosis. Int J Fertil Steril.

[10] Ruan YQ, Liang WG, Huang SH (2015). Analysis of laparoscopy on endometriosis patients with high expression of CA125. Eur Rev Med Pharmacol Sci.

[11] Liu SP, Yin XM, Wen J (2016). Serum CA125 and ALDH1 levels for diagnosis and post-operative follow-up of endometriosis. Journal of Xi’an Jiaotong University (Medical Sciences).

[12] Karimi-Zarchi M, Dehshiri-Zadeh N, Sekhavat L, Nosouhi F (2016). Correlation of CA-125 serum level and clinico-pathological characteristic of patients with endometriosis. Int JReprod Biomed.

[13] Li X-Y, Chao X-P, Leng J-H, Zhang W, Zhang J-J, Dai Y (2019). Risk factors for post-operative recurrence of ovarian endometriosis: long-term follow-up of 358 women. J Ovarian Res.

[14] Ramilo I, Alves J, Canis M, Bourdel N, Pickering G, Roman H (2014). Systematic review of endometriosis pain assessment: how to choose a scale?. Hum Reprod Update.

[15] Butrick CW (2007). Chronic pelvic pain: how many surgeries are enough?. Clin Obstet Gynecol.

[16] Ahmad MF, Narwani H, Shuhaila A (2017). An evaluation of quality of life in women with endometriosis who underwent primary surgery: a 6-month follow up in Sabah Women & Children Hospital, Sabah, Malaysia. J Obstet Gynaecol.

[17] Alborzi S, Hosseini-Nohadani A, Poordast T, Shomali Z (2017). Surgical outcomes of laparoscopic endometriosis surgery: a 6 year experience. Curr Med Res Opin.

[18] Dobrokhotova JE, Ilyina IJ, Grishin II, Ibragimova DM, Kalimatova DM, Narimanova MR (2017). Evaluation of dienogest treatment efficacy in patients with endometriosis. J Endometr Pelvic Pain Disord.

[19] Healey M, Ang WC, Cheng C (2010). Surgical treatment of endometriosis: A prospective randomised double-blinded trial comparing excision and ablation. Fertil Steril.

[20] Healey M, Cheng C, Kaur H (2014). To excise or ablate endometriosis? a prospective randomised double-blinded trial after 5-year follow-up. J Minim Invasive Gynecol.

[21] Modarresi M, Mehdizadehkashi A, Chaichian S, Ataei M, Ahmadi-Pishkuhi M (2020). Sonographic assessment of ovarian endometrioma recurrence six months after laparoscopic cystectomy in patients with endometriosis. Shiraz E Med J.

[22] Riley KA, Benton AS, Deimling TA, Kunselman AR, Harkins GJ (2019). Surgical excision versus ablation for superficial endometriosis-associated pain: a randomized controlled trial. J Minim Invasive Gynecol.

[23] Sutton CJG, Ewen SP, Whitelaw N, Haines P (1994). Prospective, randomised, double-blind, controlled trial of laser laparoscopy in the treatment of pelvic pain associated with minimal, mild, and moderate endometriosis. Fertil Steril.

[24] Abbott JA, Hawe J, Clayton RD, Garry R (2003). The effects and effectiveness of laparoscopic excision of endometriosis: a prospective study with 2–5 year follow-up. Hum Reprod.

[25] American Society for Reproductive Medicine (1997). Revised American society for reproductive medicine classification of endometriosis: 1996. Fertil Steril.

[26] Vercellini P, Fedele L, Aimi G, Pietropaolo G, Consonni D, Crosignani PG (2006). Association between endometriosis stage, lesion type, patient characteristics and severity of pelvic pain symptoms: a multivariate analysis of over 1000 patients. Hum Reprod.

[27] Jacobson TZ, Duffy JMN, Barlow DH, Koninckx PR, Garry R (2009). Laparoscopic surgery for pelvic pain associated with endometriosis. Cochrane Database Syst Rev.

